# Impact of the Timing of Endoscopic Retrograde Cholangiopancreatography for the Treatment of Acute Cholangitis: A Meta-analysis and Systematic Review

**DOI:** 10.1097/SLE.0000000000001110

**Published:** 2022-10-11

**Authors:** Yunxiao Lyu, Bin Wang, Shenjian Ye, Yunxiao Cheng

**Affiliations:** Department of Hepatobiliary Surgery, Wenzhou Medical University Affiliated Dongyang Hospital; Dongyang People’s Hospital, Dongyang, Zhejiang Province, China

**Keywords:** endoscopic retrograde cholangiopancreatography, acute cholangitis, mortality, length of hospital stay

## Abstract

The optimal timing for endoscopic retrograde cholangiopancreatography (ERCP) for acute cholangitis (AC) has not been unequivocally established.

**Aims:**

To perform a meta-analysis of the outcomes associated with particular timings of ERCP for AC.

**Methods:**

A systematic literature search was conducted for studies of ERCP for AC, and then a meta-analysis of the in-hospital mortality (IHM), 30-day mortality, and length of hospital stay (LHS) was performed.

**Results:**

Seven non-randomized studies of 88,562 patients were considered appropriate for inclusion. Compared with performing ERCP more than 24 hours after admission, ERCP within 24 hours was associated with lower IHM (*P*<0.0004), but no difference in 30-day mortality (*P*=0.38) was found between the 2 groups. ERCP performed <48 hours after admission was associated with a lower IHM and 30-day mortality (*P*<0.00001 and *P*=0.03) than ERCP performed >48 hours after admission. In addition, ERCP performed within 24 or 48 hours was associated with a shorter LHS (*P*<0.00001 and *P*<0.00001, respectively).

**Conclusion:**

ERCP within 48 hours of admission is superior to subsequent ERCP with respect to IHM, 30-day mortality, and LHS, and ERCP performed within 24 hours is associated with lower IHM and LHS.

Acute cholangitis (AC), first described by Charcot in 1877, is associated with abdominal pain, fever, and jaundice.^1^,^2^ It is an inflammatory condition that is caused by bacterial infection of the bile duct and can be life-threatening, with previous studies showing a mortality rate of between 20% and 30%.^3^,^4^ Biliary drainage is considered to be a critical step in the treatment of AC, and endoscopic retrograde cholangiopancreatography (ERCP) has become established as the gold-standard method of biliary drainage in patients with AC.^5^

The 2018 Tokyo Guidelines (TG18) recommend early ERCP for patients with AC,^6^ but the optimal timing of this early ERCP has not yet been determined. In TG18, “urgent” referred to a procedure on the day of admission and “early” referred to a procedure on the day following admission. Previous studies of the effects of the timing of ERCP on the prognosis of AC have yielded varying results. Several retrospective studies have shown that ERCP performed within 24 to 72 hours of admission is associated with lower in-hospital mortality (IHM), length of hospital stay (LHS), and 30-day mortality,^7^–^9^ whereas others did not show a difference in the mortality associated with early or late ERCP. The multi-center observational study conducted by Kiriyama et al^10^ in Japan and Taiwan showed that ERCP performed within 24 or 48 hours of admission was not superior to later procedures with respect to 30-day mortality.

The existing literature is thus equivocal regarding whether early ERCP for AC is associated with superior outcomes to later ERCP. From a clinical perspective, it would be of great interest to clarify any relationship between the timing of ERCP and the mortality rate of patients with AC. Therefore, we conducted a meta-analysis and systematic review to evaluate the existing evidence regarding the effect of the timing of ERCP on the outcomes of AC.

## MATERIALS AND METHODS

### Data Sources

The present meta-analysis is reported according to the Preferred Reporting Items for Systematic Reviews and Meta-Analyses^11^ and the Cochrane Handbook for Systematic Reviews of Interventions.^12^ Two authors independently performed a thorough electronic search of the PubMed, Embase, Web of Science, Cochrane Central Register of Controlled Trials, and ClinicalTrials.gov databases for records up to February 1, 2022. Original studies of the timing of ERCP for AC were included. The English search terms included, but were not limited to, the following: “ERCP,” “acute cholangitis,” “endoscopic retrograde cholangiopancreatography,” and “cholangitis”. The search was restricted to human subjects and English-language articles. The reference lists of the articles identified in the initial search were also manually reviewed.

### Inclusion and Exclusion Criteria

Only comparative studies (ERCP <24 h vs. ERCP >24 h or ERCP <48 h vs. ERCP >48 h after admission) of ERCP for AC were included. The included studies were required to provide a clear definition of acute cholangitis, and that included studies that compared patients with all grades of cholangitis. Review articles, abstracts, and case reports were excluded.

### Data Extraction and Outcome Measures

Two authors extracted the data from the included studies using the following standardized format: first author, year and country of publication, study design, sample size, etiology of AC, and reported outcomes. The outcomes of the present study were IHM, 30-day mortality and LHS disparities in the data extracted were resolved by discussion and by reference to the original article. EndNote version X8 (Thomson Reuters was used to remove duplicate studies.

### Quality Assessment

The Newcastle-Ottawa quality assessment scale was used to evaluate the quality of cohort studies in 3 areas: the recruitment of cases and controls, the comparability of the 2 groups, and the outcomes of interest.^13^

### Statistical Analysis

Statistical analyses were performed using Review Manager (RevMan) version 5.3 software (Cochrane Informatics and Knowledge Management Department, Nordic Cochrane Centre). Odds ratios (ORs) with 95% confidence intervals (CIs) were calculated for dichotomous outcomes. Publication bias was evaluated using the χ^2^ test and funnel plots. Heterogeneity among the studies was evaluated using the χ^2^ test. A 2-tailed *P* value of <0.05 was considered to represent statistical significance. We also assessed the potential for publication bias through a visual inspection of the funnel plots for asymmetry.

## RESULTS

### Study Selection and Trial Characteristics

**FIGURE 1:**
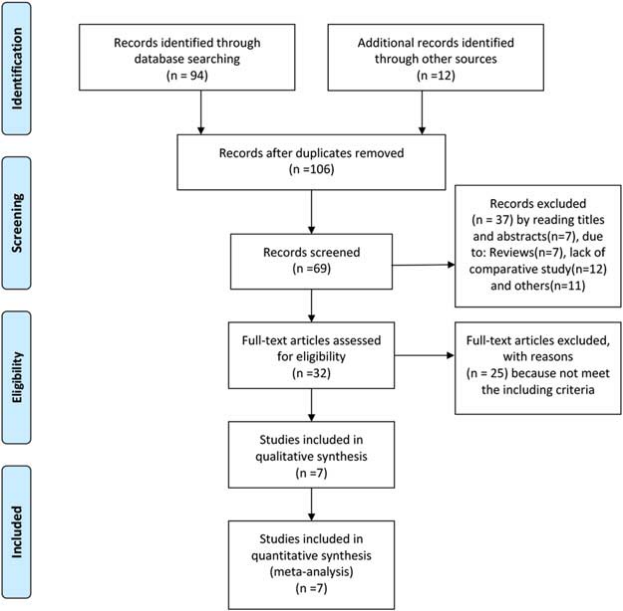
Flow diagram of the published articles that were evaluated for inclusion in the present meta-analysis.

Of the 106 articles selected based on their titles, 37 were excluded because they were abstracts (27), reviews (7), not comparative studies (12), or for other reasons (11). After reading the full text of each article, a further 25 were excluded because they did not meet the inclusion criteria. In addition, the study conducted by Park and colleagues were excluded because it was performed in patients of >75 years of age, and 2 other studies that focused on either nonsevere AC or severe AC were also excluded. Finally, 7 articles met the inclusion criteria, and the data within were included in the present meta-analysis.^8^,^10^,^14^–^18^ A flow chart of the literature search process is shown as Figure 1.

The characteristics and quality evaluation of the included studies are shown in Table 1. The year of publication of the included studies ranged between 2000 and 2019, and the studies were conducted in Japan, United States, and Denmark. Two of the studies were database studies and 5 were retrospective studies. Four of the studies compared ERCP <48 hours and >48 hours after admission because of AC, 1 of the studies compared ERCP <24 hours and >24 hours, and 2 compared ERCP <24 hours, 24 to 48 hours, and >48 hours after admission. The etiologies of AC included cholelithiasis, malignancy, primary sclerosing cholangitis, and benign stricture. The IHM and 30-day mortality of the participants were reported in 4 articles.

**TABLE 1:** Characteristics of Included Studies

References	Country	Study period	Study design	Sample size	Age (y)	Sex (M/F)	Etiology of acute cholangitis	Outcomes	NOS score
Kiriyama et al^10^	Japan	January 2011 to December 2012	Retrospective study	ERCP <48 h N=3730 ERCP ≥48 h N=2333	NR	NR	Bile duct stones 3659 Malignancy 948 Stent obstruction 667 Others 671 Unknown 386	30-day mortality	7
Lee et al^8^	USA	April 2005 to March 2013	Retrospective study	ERCP <48 h N=126 ERCP ≥48 h N=77	59 (18) 59 (21)	52/74 40/37	Bile duct stones 115 Malignancy 40 Stent obstruction 30 Others 18	Persistent organ failure Acute renal failure Mechanical ventilation Hypotension Intensive care unit stay Length of hospital stay In-hospital mortality 30-day mortality	8
Mulki^15^	USA	2014	Database Study	ERCP <48 h N=3042 ERCP ≥48 h N=1528	63.6 (18.1) 65.1 (18.3)	1485 717	Bile duct stones 3061 Malignancy 770 others 739	Length of stay in days Hospitalization costs in-hospital mortality 30-day mortality 30-day readmissions	7
Navaneethan et al^16^	USA	January 2001 to August 2012	Retrospective study	ERCP <48 h N=127 ERCP ≥48 h N=41	63 (52 to 74) 61 (46.5 to 70)	70/57 23/18	Bile duct stones 65 Malignancy 28 Primary sclerosing Cholangitis 45 Benign stricture 20 Others 10	Length of hospital stay After-ERCP adverse event	6
Parikh et al^17^	USA	1998 to 2012	Database Study	ERCP <24 h N=45733 ERCP 24 h-48 h N=13916 ERCP>48 h N=17674	69.1 (0.23) 70.2 (0.35) 72.0 (0.36)	20896/24837 6392/7524 8101/9573	Choledocholithiasis	Length of stay In-hospital mortality	7
Patel 18	USA	January 2009 to August 2012	Retrospective study	ERCP <24 h N=23 ERCP 24 h-48 h N=12 ERCP >48 h N=34	60 (25) 41 (18) 55 (17)	9/14 4/8 15/19	CBD stone/sluge 44 Benign stricture 10 Malignancy 11 Stent occulusion 4	In-hospital mortality Length of stay	6
Tan [14]	Denmark	March 2009 to September 2016	Retrospective study	ERCP <24 h N=48 ERCP ≥24 h N=118	65±11 73±8	25/23 67/51	Bile duct stones 74 Malignancy 66 Stent obstruction 13 Others 13	30-day mortality Organ failure Length of hospital stay Intensive care unit stay	7

ERCP indicates endoscopic retrograde cholangiopancreatography.

### Outcomes of ERCP <24 Hours Versus >24 Hours After Admission

#### In-hospital Mortality

**FIGURE 2:**
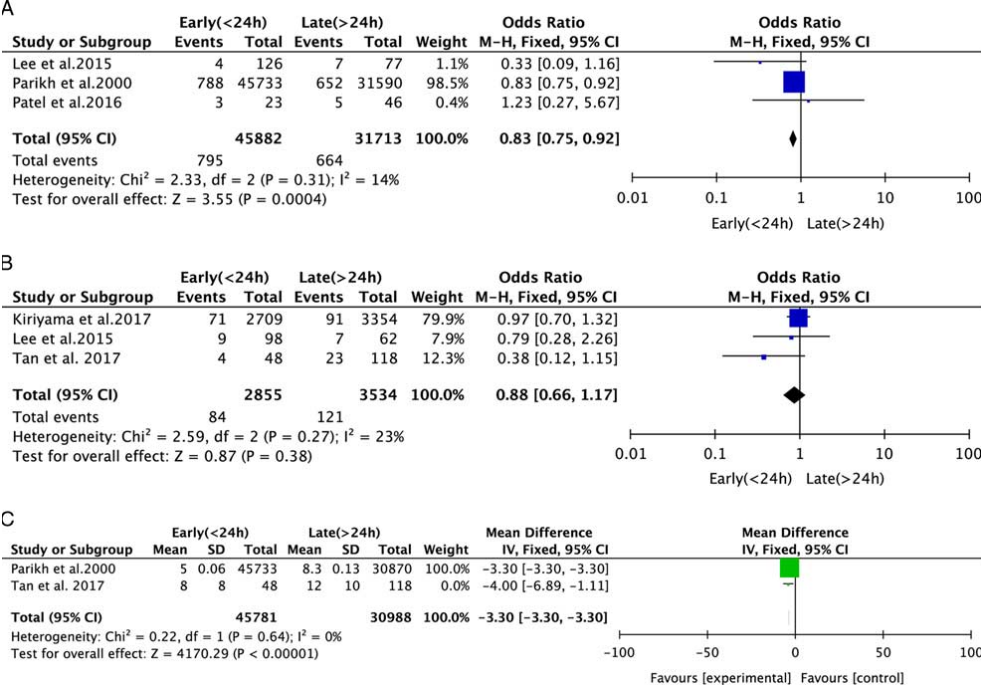
Forest plot of the meta-analysis of ERCP performed <24 hours and >24 hours after admission. A, In-hospital mortality; B, 30-day mortality; C, Length of hospital stay.

Three studies of 82,165 participants compared patients who underwent ERCP less and more than 24 hours after admission (<24 h, n=45,882; >24 h, n=31,713). Using fixed effect mode, we found that ERCP within 24 hours was associated with lower IHM than ERCP >24 hours after admission (OR 0.83, 95% CI 0.75–0.92 *P*<0.0004) (Fig. 2A). We performed a sensitivity analysis by removing 1 study at a time from the analysis, and we found that there was no longer a significant difference between the 2 groups (OR 0.56, 95% CI 0.21–1.47; *P*=0.24) when we removed the study conducted by Parikh et al.^17^

#### 30-day Mortality

Four studies of 6389 participants compared ERCP performed <24 hours (n=2855) and >24 hours (n=3534) after admission. There was no significant difference between the 2 groups in terms of 30-day mortality (OR 0.88, 95% CI 0.66 to 1.17 *P*=0.38) (Fig. 2B). Sensitivity analysis showed that this finding remained when each study was removed in turn.

#### Length of Hospital Stay

Three studies provided data regarding LHS. ERCP performed within 24 hours was associated with a shorter LHS than ERCP performed after 24 hours (mean difference−3.30, 95% CI −3.30 to −3.30; *P*<0.00001) (Fig. 2C). The finding remained when each study was removed in turn.

### ERCP <48 Hours Versus >48 Hours After Admission

#### In-hospital Mortality

**FIGURE 3:**
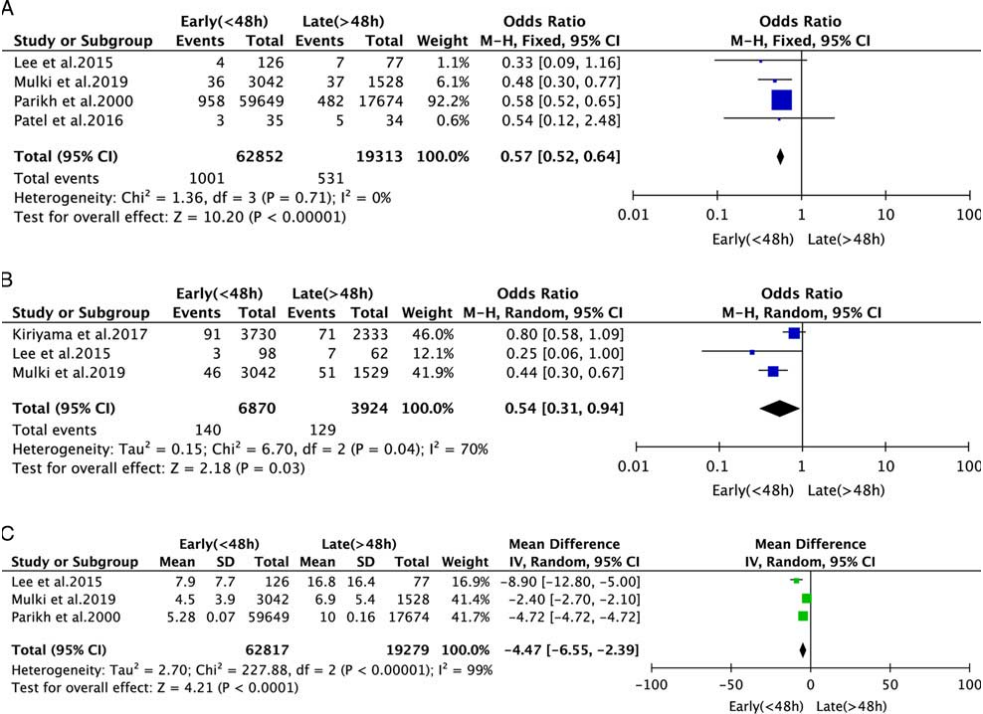
Forest plot of the meta-analysis of ERCP performed <48 hours and >48 hours after admission. A, In-hospital mortality; B, 30-day mortality; C, Length of hospital stay.

Three studies of 82,165 participants provided data regarding IHM for ERCP performed <48 hours and >48 hours after admission. We found that ERCP after <48 hours was associated with lower IHM (OR 0.57, 95% CI 0.52 to 0.64 *P*<0.00001) (Fig. 3A), and this finding remained when we removed each study from the analysis in turn.

#### 30-day Mortality

Three studies of 10,794 participants compared the 30-day mortality associated with ERCP performed <48 hours (n=6870) and >48 hours (n=3924) after admission. ERCP performed within 48 hours was associated with lower 30-day mortality (OR 0.54, 95% CI 0.31 to 0.94; *P*=0.03) (Fig. 3B), and the result remained when each study was removed in turn.

#### Length of Hospital Stay

Three studies provided data regarding LHS. ERCP performed within 48 hours was associated with a shorter stay (MD −4.48% CI −6.55% to −2.39% *P*<0.00001) (Fig. 3C)., and the result remained when each study was removed in turn.

## DISCUSSION

The aim of the present meta-analysis was to evaluate the effect of the timing of ERCP on the outcomes of AC. We found that ERCP performed within 24 hours of admission was not superior with respect to IHM or 30-day mortality and that ERCP performed within 48 hours had an advantage with respect to IHM, but not 30-day mortality. However, ERCP performed within 24 or 48 hours of admission was associated with a shorter hospital stay.

Previous studies have suggested that the earlier recognition of cholangitis and more widespread use of biliary drainage could reduce the overall mortality associated with AC.^19^ Several previous guidelines have also mentioned the timing of endoscopic biliary drainage for patients with AC. For example, the guidelines published by the European Society of Gastrointestinal Endoscopy recommend that ERCP for AC should be timed according to the severity of AC, ranging from 12 to 72 hours.^20^ However, the guidelines published by the American Society for Gastrointestinal Endoscopy and the TG18 did not make specific recommendations regarding the timing of ERCP.^21^ TG18 defined “urgent” ERCP as being on the day of admission (within 24 h) and “early” ERCP as being on the day following admission (24 to 48 h).^6^ Thus, the optimal timing of endoscopic biliary drainage for AC has not been established.

In the present study, we found that ERCP performed within 24 hours was associated with lower IHM. Of note, if the study by Parikh and colleagues was removed from the analysis, ERCP within 24 hours was not superior to ERCP performed subsequently. However, because this study comprised 77,323 participants,^17^ whereas the other 2 studies were small, we believe that the inclusion of this study is necessary and that ERCP performed within 24 hours of admission can reduce IHM. Notably, the study by Parikh and colleagues only included patients with choledocholithiasis.

With respect to 30-day mortality, we found no significant difference between ERCP performed <24 hours and >24 hours after admission. This finding is consistent with those of several previous studies. A study conducted by Patel et al^18^ demonstrated that the timing of ERCP was not associated with mortality, irrespective of the severity of AC. Aboelsoud et al^22^ conducted a study that included critically ill patients and showed that ERCP performed within 24 hours did not reduce IHM or 28-day mortality, although it was associated with a lower incidence of persistent organ failure and shorter ICU stays. Nonetheless, few studies have compared ERCP performed <24 hours and >24 hours after admission. The heterogeneity of the comparison of ERCP performed <24 hours and >24 hours after admission was low, which showed the conclusion was stability. Nevertheless, more high-quality research is needed in the future.

In the present study, when ERCP > 48 hours and <48 hours after admission were compared, we found that earlier ERCP was associated with lower IHM and 30-day mortality, consistent with the findings of most previous studies. A cohort study of 203 patients showed that ERCP > 48 hours after admission was associated with persistent organ failure, whereas ERCP < 48 hours after admission was associated with lower IHM and a shorter hospital stay.^8^ Another nationwide analysis showed that early ERCP (<48 h) was associated with lower IHM and 30-day mortality. However, because of a lack of consistent reporting standards, we were unable to collect sufficient data regarding the complication rates associated with ERCP to analyze this outcome. In addition, several studies were excluded from the present analysis.^15^ A recent study that compared patients who underwent ERCP <12 versus >12 hours and <6 versus >6 hours after admission showed that ERCP neither <6 nor <12 hours after admission was associated with superior clinical outcomes but may result in a lower incidence of rehospitalization.^23^ Another 2 studies were excluded because they included patients with nonsevere cholangitis.^24^,^25^ Thus, the relationship between the timing of ERCP and the severity of cholangitis is also unclear, and more research is required to address this question. It is worth noting that regional differences may lead to differences in the timing of ERCP for acute cholangitis. European Society of Gastrointestinal Endoscopy recommends that ERCP could be performed from 12 to 72 hours. However, the guideline of American Society for Gastrointestinal Endoscopy and TG18 did not make specific recommendations regarding the timing of ERCP.

The present study had several limitations. First, the studies included in the meta-analysis were retrospective or based on databases, which may have resulted in selection bias. The characteristics of the patients included in the database study varied significantly. The etiology of AC was variable in the included studies. Second, the etiologies of AC in the included studies varied, which may also have introduced bias. Third, the outcomes reported in the included studies varied. There was a lack of data regarding the adverse events associated with ERCP, and therefore we could not include these in the meta-analysis. In addition, the definition of time varies in the included literature. Because of these shortcomings, a larger, high-quality randomized control trial regarding the timing of ERCP should be conducted in the future.

In conclusion, ERCP performed within 48 hours of admission has advantages with respect to the IHM, 30-day mortality, and LHS associated with AC over ERCP performed later. In addition, ERCP performed within 24 hours is associated with lower IHM and LHS.
